# Chronic diseases spectrum and multimorbidity in elderly inpatients based on a 12-year epidemiological survey in China

**DOI:** 10.1186/s12889-024-18006-x

**Published:** 2024-02-17

**Authors:** Shan Gao, Shasha Sun, Ting Sun, Tingting Lu, Yan Ma, Hebin Che, Miao Liu, Wanguo Xue, Kunlun He, Yabin Wang, Feng Cao

**Affiliations:** 1 Chinese PLA Medical School, Chinese PLA General Hospital, 28# Fuxing Road, Beijing, Haidian District 100853 China; 2 Department of Cardiology & National Clinical Research Center for Geriatric Diseases, The Second Medical Center of Chinese PLA General Hospital, 28# Fuxing Road, Beijing, Haidian District 100853 China; 3 Medical Big Data Research Center, Chinese PLA General Hospital, 28# Fuxing Road, Beijing, Haidian District 100853 China; 4 State Key Laboratory of Kidney Disease, Chinese PLA General Hospital, 28# Fuxing Road, Beijing, Haidian District 100853 China

**Keywords:** Disease spectrum, Multimorbidity, Polypharmacy, Elderly

## Abstract

**Background:**

The number and proportion of the elderly population have been continuously increasing in China, leading to the elevated prevalence of chronic diseases and multimorbidity, which ultimately brings heavy burden to society and families. Meanwhile, the status of multimorbidity tends to be more complex in elderly inpatients than community population. In view of the above concerns, this study was designed to investigate the health status of elderly inpatients by analyzing clinical data in Chinese People's Liberation Army (PLA) General Hospital from 2008 to 2019, including the constitution of common diseases, comorbidities, the status of multimorbidity, in-hospital death and polypharmacy among elderly inpatients, so as to better understand the diseases spectrum and multimorbidity of elderly inpatients and also to provide supporting evidence for targeted management of chronic diseases in the elderly.

**Methods:**

A clinical inpatients database was set up by collecting medical records of elderly inpatients from 2008 to 2019 in Chinese PLA General Hospital, focusing on diseases spectrum and characteristics of elderly inpatients. In this study, we collected data of inpatients aged ≥ 65 years old, and further analyzed the constitution of diseases, multimorbidity rates and mortality causes in the past decade. In addition, the prescriptions were also analyzed to investigate the status of polypharmacy in elderly inpatients.

**Results:**

A total of 210,169 elderly patients were hospitalized from January 1st, 2008 to December 31st, 2019. The corresponding number of hospitalizations was 290,833. The average age of the study population was 72.67 years old. Of the total population, 73,493 elderly patients were re-admitted within one year, with the re-hospitalization rate of 25.27%. Malignant tumor, hypertension, ischemic heart disease, diabetes mellitus and cerebrovascular disease were the top 5 diseases. Among the study population, the number of patients with two or more long-term health conditions was 267,259, accounting for 91.89%, with an average of 4.68 diseases. In addition, the average number of medications taken by the study population was 5.4, among which, the proportion of patients taking more than 5 types of medications accounted for 55.42%.

**Conclusions:**

By analyzing the constitution of diseases and multimorbidity, we found that multimorbidity has turned out to be a prominent problem in elderly inpatients, greatly affecting the process of healthy aging and increasing the burden on families and society. Therefore, multidisciplinary treatment should be strengthened to make reasonable preventive and therapeutic strategies to improve the life quality of the elderly. Meanwhile, more attention should be paid to reasonable medications for elderly patients with multimorbidity to avoid preventable side effects caused by irrational medication therapy.

**Supplementary Information:**

The online version contains supplementary material available at 10.1186/s12889-024-18006-x.

## Introduction

Population aging has been steadily increasing worldwide. By the end of 2022, there were 209.78 million people aged 65 or above in China, accounting for 14.9% of the total population. Meanwhile, it is estimated that in the year 2057, the number of elderly will reach a peak at 425 million, accounting for 32.9%-37.6% of the national population [1, 2]. Since aging is accompanied by a decreased reserve function of multiple organs as well as an increased prevalence of multiple chronic diseases, the elderly can be challenged to maintain good health and quality of life [3, 4].

Multimorbidity, defined as the presence of two or more long-term physical or mental health conditions, which cannot be currently cured but can be improved with multidisciplinary management, has been an increasingly prominent issue associated with population aging [5–7]. A previous meta-analysis showed that the prevalence of multimorbidity widely varied across studies, around 20–30% when the whole population was considered but from 55 to 98% when only older persons were included [8]. The prevalence of multimorbidity increases with age and varies widely across studies. A previous systematic review revealed that there was an S-shaped curve for the association between age and prevalence of multimorbidity. Before the age of 40 years, the prevalence was about 20% or lower and then increased dramatically, which eventually reach a plateau of 75% at the age of 70 years [9]. Another study focusing on the mortality related to multimorbidity revealed that patients with multimorbidity had a 37% increased risk of death [10]. Compared to patients with a single condition, multimorbidity patients have an increased risk of functional decline, poorer quality of life, longer hospital stay, and increased healthcare costs [11, 12]. In addition, several studies on patterns of multimorbidity showed that hypertensive diseases were the most frequent condition in multimorbidity pattern, followed by diabetes mellitus and arthropathies, and the most frequent single disease conditions identified in patients with multimorbidity were hypertensive diseases, diabetes mellitus, cerebrovascular diseases, heart diseases, arthropathies, and chronic lower respiratory diseases [13].

Additionally, as the number of diseases increases, the number of drug prescriptions tends to increase. Polypharmacy, which is common among elderly patients with multimorbidity, is stated as the concurrent use of multiple medications [14]. While there is no consensus on the medication threshold, polypharmacy is often commonly defined as concomitant use of 5 or more medications [15, 16]. Previous studies have revealed that polypharmacy was associated with numerous poor health outcomes, including an increased risk of hospitalization and death, which was probably related to drug interactions and non-adherence [15, 17, 18].

Despite multimorbidity is strongly associated with premature death, higher hospitalization rate, worse health outcomes and higher healthcare costs, most guidelines on the management of patients with multimorbidity remain focusing on single diseases [19, 20]. To date, a previous study has focused on the patterns of multimorbidity to recognize associations between common diseases in a longitudinal study among Chinese community population [21]. However, the comprehensive analysis of diseases spectrum, multimorbidity, polypharmacy, organ dysfunction and mortality of elderly inpatients is relatively scarce. Therefore, it is urgent to conduct a comprehensive investigation on the profile of common diseases and multimorbidity of elderly inpatients, so as to guide the rational allocation of limited medical resources to meet the healthcare needs of the elderly.

In this study, we collected the clinical data of elderly inpatients aged ≥ 65 years in Chinese PLA General Hospital from 2008 to 2019, and further analyzed the constitution of common diseases, comorbidities, and the status of multimorbidity of the study population. In addition, we collected oral drug prescriptions and analyzed the oral medications taken by the study population to clarify the profile of polypharmacy in the elderly inpatients, ultimately aiming to provide supporting evidence for the comprehensive management of the elderly inpatients.

## Methods

### Study design and participants

The study was designed as a full-sampled survey recruiting 210,169 patients who were hospitalized in Chinese PLA General Hospital from January 1st, 2008 to December 31st, 2019. The Inclusion and exclusion criteria are listed below: Inclusion criteria: (1) elderly patients aged ≥ 65 years old; (2) patients hospitalized in Chinese PLA General Hospital from 2008 to 2019; (3) patients with at least one type of chronic disease based on discharge diagnoses. Exclusion criteria: (1) patients with incomplete data of age or gender; (2) patients with incomplete data of discharge diagnosis; (3) patients with incomplete data of medications;(4) patients without any type of chronic disease. The study has been approved by the Ethics Committee of Chinese PLA General Hospital (Unique identifier: S2022-522–01). Meanwhile, all participants signed informed consent as required.

### Data collection

In this study, we collected all the data through the big data platform of PLA General Hospital, the construction of which was based on the original case data of patients in the hospital from 2000 to 2019. This work was supported by the Medical Big Data Research Center of PLA General Hospital. The variables we focused on include demographic data (age and gender), discharge diagnoses (major diagnoses and other diagnoses), causes of in-hospital death, and drug prescriptions.

### Statistical analysis

Firstly, we assessed the annual changes in the number of elderly inpatients and hospitalizations. Besides, the demographic data were analyzed. Secondly, we analyzed all items of discharge diagnoses according to the International Classification of Diseases (ICD–10), and assessed the number of patients with specific health conditions, so as to explore the diseases spectrum of the study population. Hereon, the proportion of elderly inpatients with one of the most prevalent diseases (the number of elderly inpatients with one of the most prevalent diseases/the total number of elderly inpatients) was the index to evaluate the ranking of various diseases in analysis. Multiple types of organ failures, which are commonly developed in the elderly due to reduced organ compensatory function, were specifically counted and analyzed in this study. Since the occurrence of organ failure is a functional state associated with a variety of diseases in most cases, rather than an exact disease, we counted once if an item of diagnosis of any type of organ failure occurred in one admission time. Later, we analyzed the most prevalent diseases in multiple age and sex groups to clarify the variations of diseases spectrum in various age and sex groups.

Meanwhile, we specifically assessed the items of first diagnoses to show the reasons for hospitalizations of the study population. We further focused on prevalence and patterns of multimorbidity among the study population, including incidence and distribution of comorbidities. Additionally, we focused on the elderly inpatients in whom we observed in-hospital death, and analyzed the composition of death causes. Last but not least, we analyzed the most commonly used oral medications in the study population and grouped them according to pharmacological effects, mainly including calcium channel blockers (CCB), statins, aspirin, beta-adrenergic blockers, nitrates, angiotensin receptor blockers (ARB) and other types of medications, aiming to illustrate the status of polypharmacy in the study population. Hereon, the proportion of patients applying one of the most used medications was the index to evaluate the ranking of medications.

Since the study was a full-sampled survey, the sample size was 210,169 which was based on the total number of elderly inpatients who met the inclusion and exclusion criteria listed above. Continuous data were presented as either mean ± SD for normal distribution data or medians (IQRs) for non-normal distribution data. Meanwhile, categorical variables were presented as frequencies (percentages) in our study. All data were statistically analyzed with SPSS 26.0 (SPSS Inc., IBM, Chicago, IL, USA). The number of hospitalizations was used as the index to show the results. 

## Results

### Demographic characteristics of the study population

**Fig. 1:**
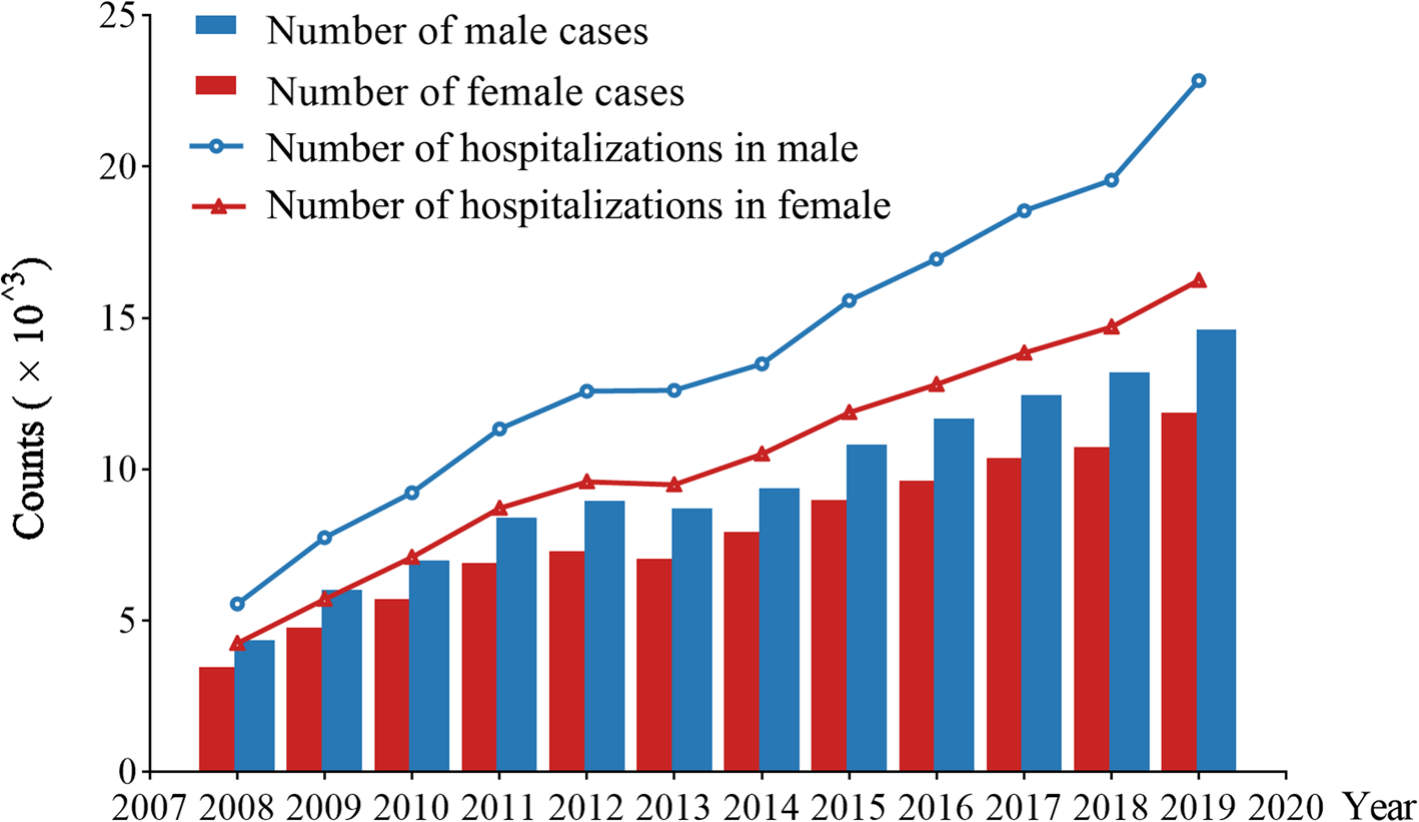
The number of inpatients and hospitalizations from 2008 to 2019. Columns in blue represent the number of male inpatients, while those in red represent the number of female inpatients in each year from 2008 to 2019. The line in blue represents the variation of hospitalizations in males, while that in red represents the variation of hospitalizations in females from 2008 to 2019

**Fig. 2:**
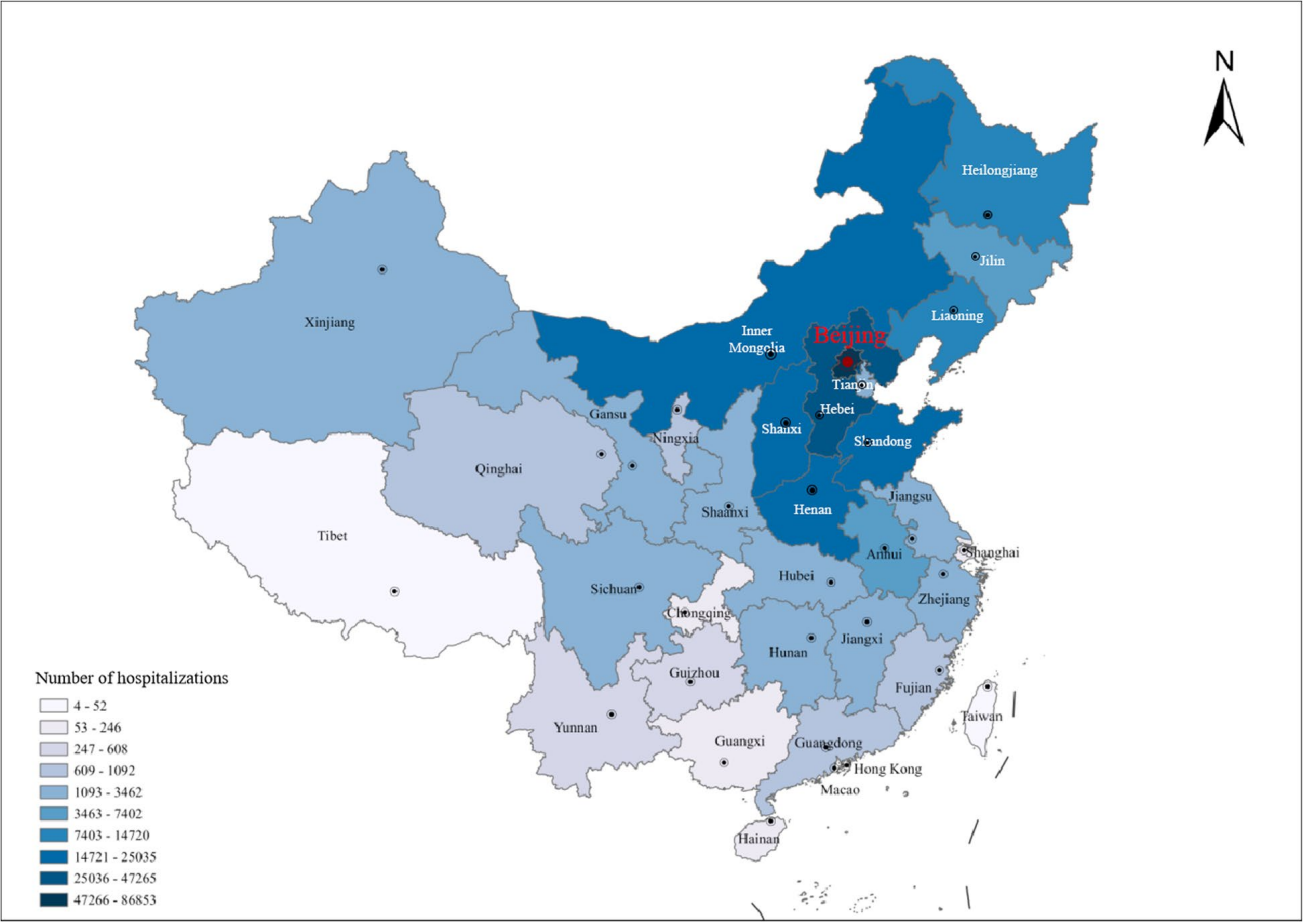
The regional distribution of the study population

A total of 210,169 elderly patients (115,541 male and 946,28 female, respectively), hospitalized in Chinese PLA General Hospital from 2008 to 2019, were enrolled in this study and the total number of hospitalizations was 290,833 (165,983 male and 124,850 female, respectively). Meanwhile, we found that the number of inpatients and the number of hospitalizations each year from 2008 to 2019 both showed overall upward trends (Fig. 1). The characteristics of the study population were listed in Table 1. The average age of the study population was 72.67 ± 5.55 years, of which the maximum age was 120 years old. Meanwhile, the age composition of the study population was shown in Fig.S1. The total number of cases who were re-admitted to the hospital within one year was 73,493, with a one-year readmission rate of 25.27%. It is worth noting that 267,259 (91.89%) patients were diagnosed with two or more types of long-term health conditions. In addition, we also counted the geographical distribution with the results presented in Fig. 2, indicating that the elderly inpatients in our study were from 34 provincial-level administrative regions in China, mainly from the north, east and northeast regions of China.

**Table 1:** Characteristics of the study population

Characteristic	Value
Male, n(%)	165,983 (57.07%)
Age (years)	72.67 ± 5.55
Total number of readmission within one year, n(%)	73,493 (25.27%)
Number of patients with two or more types of long-term conditions, n(%)	267,259 (91.89%)
The number of patients with various numbers of diseases, n(%)	
1	23,574 (8.11%)
2	35,893 (13.43%)
3	21,728 (8.13%)
4	30,868 (11.55%)
5	34,690 (12.98%)
6	30,895 (11.56%)
7	25,630 (9.59%)
8	24,561 (9.19%)
9	12,802 (4.79%)
≥ 10	28,543 (10.68%)

All data were counted according to the number of hospitalizations. Data given as n (%) unless otherwise indicated. Proportions calculated using the total number of each item as the denominator. Age was expressed as mean ± SD, standard deviation

### The disease distribution in the elderly inpatients

#### The composition of chronic diseases in the study population

**Fig. 3:**
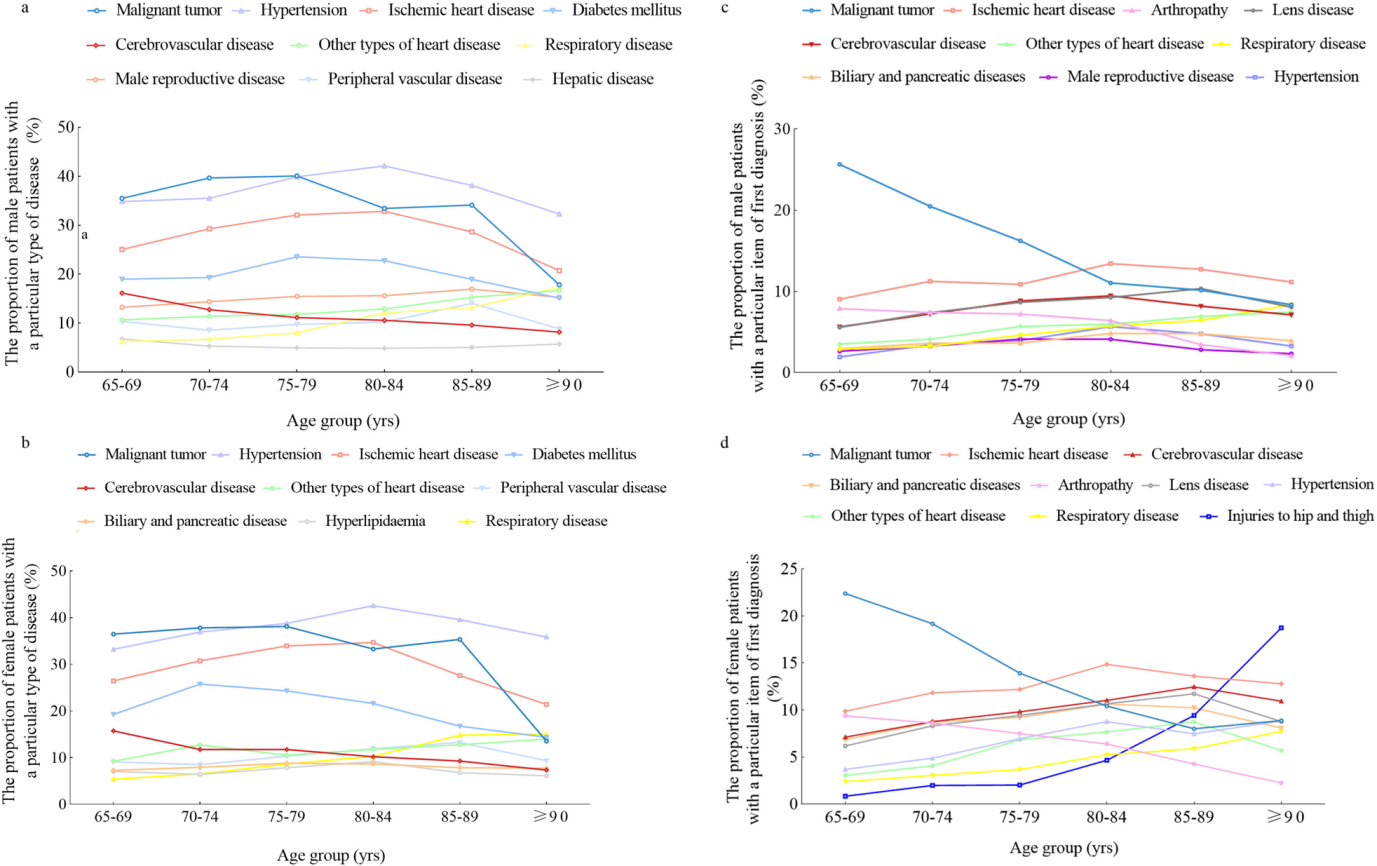
The proportion of top 10 diseases and top 10 causes of hospitalization in the study population. (**a**) the proportion of top 10 diseases of various age groups in male elderly inpatients (**b**) the proportion of top 10 diseases of various age groups in female elderly inpatients (**c**) The proportion of top 10 causes of hospitalization of various age groups in male elderly patients (**d**) The proportion of top 10 causes of hospitalization of various age groups in female elderly inpatients

We analyzed the diseases spectrum among the study population and found that malignant tumor, hypertension, ischemic heart disease, diabetes mellitus, cerebrovascular disease, other types of heart disease, peripheral vascular disease, biliary and pancreatic disease, hyperlipidemia and respiratory disease were the most prevalent diseases in the study population, which was shown in Table 2. Given the differences between men and women, including physiological features, psychological factors, family roles, social environment and so on, we counted and analyzed disease distribution among male and female elderly inpatients separately. We found that malignant tumor (37.36%), hypertension (36.76%), ischemic heart disease (28.51%), diabetes mellitus (20.28%), cerebrovascular disease (13.38%), other types of heart disease (11.50%), male reproductive disease (14.36%), peripheral vascular disease (9.77%), respiratory disease (7.52%) and hepatic disease (5.74%) were the top 10 diseases in male elderly inpatients. In females, the ranking of top 6 diseases was the same as that of males (malignant tumor (36.72%), hypertension (36.59%), ischemic heart disease (30.06%), diabetes mellitus (22.41%), cerebrovascular disease (12.90%) and other types of heart disease (10.97%). Peripheral vascular disease (9.56%), biliary and pancreatic disease (7.95%), hyperlipidemia (7.21%) and respiratory disease (7.19%) ranked 7th to 10th in females. In addition, we further analyzed the disease composition of various age groups both in males and females based on the age distribution of the study population, and the results were shown in Fig. 3. In both male and female elderly inpatients, the proportion of malignant tumor increased first and then decreased with age, while other types of heart disease and respiratory disease presented an upward trend gradually.

**Table 2:** The diseases composition and causes of hospitalization in the study population

Items	Proportion
Composition of diseases	
Malignant tumor	37.18%
Hypertension	36.69%
Ischemic heart disease	29.18%
Diabetes mellitus	20.75%
Cerebrovascular disease	13.19%
Other types of heart disease	11.27%
Peripheral vascular disease	9.68%
Biliary and pancreatic disease	7.62%
Hyperlipidemia	7.45%
Respiratory disease	7.39%
Causes of hospitalization	
Malignant tumor	19.34%
Ischemic heart disease	11.01%
Cerebrovascular disease	7.85%
Arthropathy	7.69%
Lens disease	7.51%
Biliary and pancreatic disease	5.63%
Other types of heart disease	4.59%
Hypertension	4.13%
Peripheral vascular disease	3.76%
Esophagus, stomach and duodenum disease	3.11%

All data were counted according to the number of hospitalizations. Data was given as proportions unless otherwise indicated. Proportions were calculated using the total number of each item as the denominator

#### The composition of organ dysfunctions in the study population

Of 7963 items of single organ failure diagnosis recorded in the data, 3293 (41.36%) were heart failure, 3406 (42.77%) were renal failure, 1030 (12.93%) were respiratory failure and 234 (2.94%) were hepatic failure. In addition, 5315 patients were diagnosed with two types of organ failures, including heart failure combined with renal failure (53.76%), heart failure combined with respiratory failure (17.81%), respiratory failure combined with renal failure (11.87%), hepatic failure combined with renal failure (6.63%), heart failure combined with hepatic failure (4.67%) and respiratory failure combined with hepatic failure (5.26%). All the results on the composition of single and two types of organ failure were shown in Table S1.

### The causes of hospitalization in the study population

In order to figure out frequent causes of hospitalization in elderly inpatients, we analyzed the first item of discharge diagnoses separately. It was found that malignant tumor, ischemic heart disease, cerebrovascular disease, arthropathy, lens disease, biliary and pancreatic disease, other types of heart disease, hypertension, peripheral vascular disease, and esophagus, stomach and duodenum disease were the top 10 causes of hospitalization in the study population (Table 2). When counted by sex, we found that malignant tumor (20.36%), ischemic heart disease (10.61%), arthropathy (7.32%), lens disease (7.23%), cerebrovascular disease (7.18%), other types of heart disease (4.47%), respiratory disease (3.73%), biliary and pancreatic disease (3.53%), male reproductive disease (3.26%) hypertension (3.21%), were the top 10 diseases related to hospitalizations in elderly male inpatients. While malignant tumor (17.99%), ischemic heart disease (11.55%), cerebrovascular disease (8.73%), biliary and pancreatic disease (8.37%), arthropathy (8.26%), lens disease (8.11%), hypertension (5.35%), other types of heart disease (4.76%), respiratory disease (3.26%) and injuries to hip and thigh (2.19%) ranked top 10 in females. Later, we further analyzed the differences between different gender and age groups (Fig. 3) and found that the proportions of malignant tumor declined with age in both male and female groups. Moreover, it is worth noting that in female inpatients, the proportion of arthropathy showed a downward trend while injuries to hip and thigh presented an upward trend.

### The status of multimorbidity in the study population

#### The general condition of multimorbidity in the study population

**Fig. 4:**
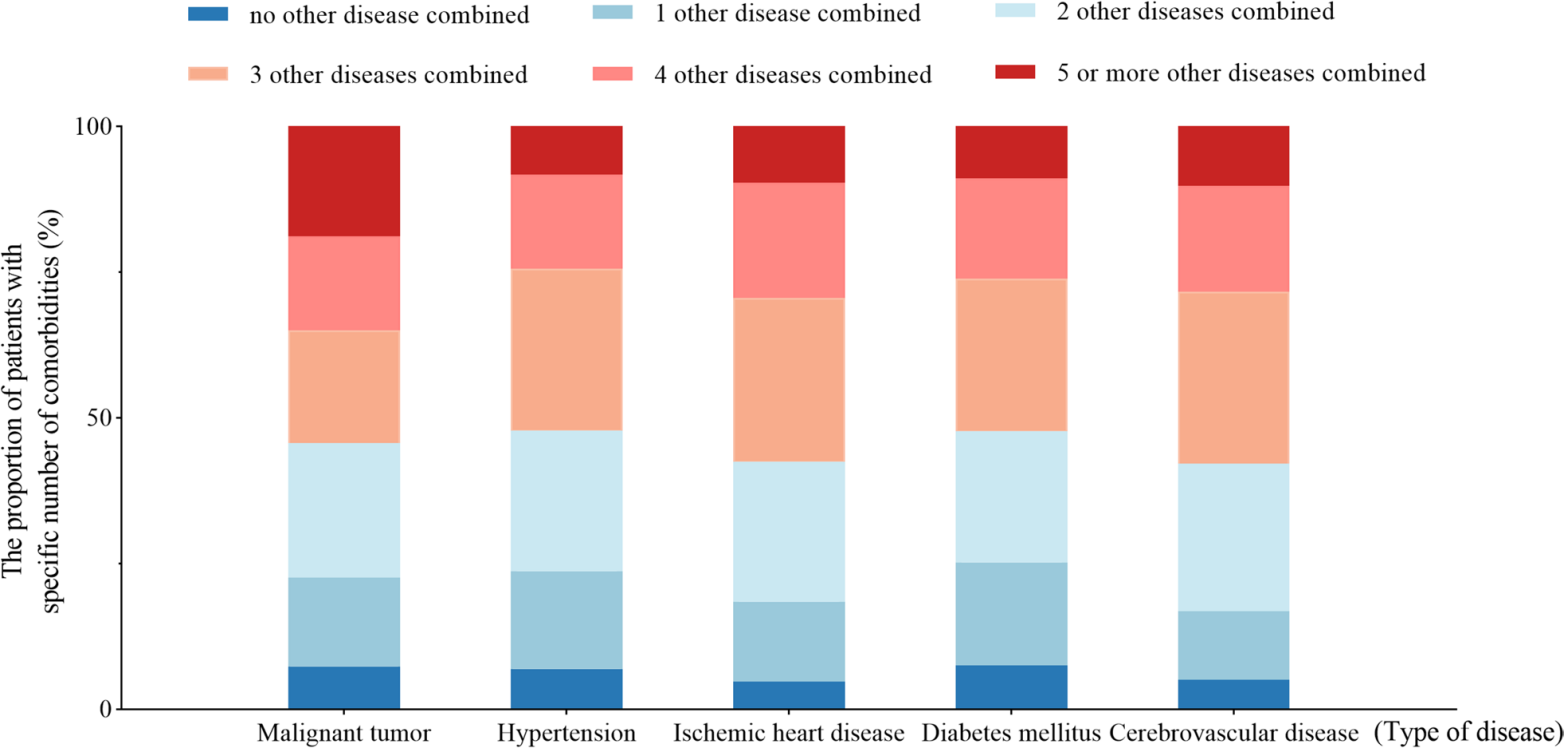
The composition of comorbidities of top 5 diseases in the study population. Malignant tumor, hypertension, ischemic heart disease, diabetes mellitus and cerebrovascular disease are the top 5 diseases in the study population according to the results in the section of the disease distribution in the elderly inpatients. Therefore, we further analyzed the composition of comorbidities in patients with the top 5 diseases

Among the study population, the number of patients with two or more long-term health conditions was 267,259, accounting for 91.89%, with an average of 4.68 kinds of diseases. The proportion of patients with various numbers of diseases was shown in Fig. S2, indicating that the coexistence of multiple chronic diseases is prominent in elderly inpatients. We further analyzed the number of comorbidities among patients with the top 5 diseases listed above, including malignant tumor, hypertension, ischemic heart disease, diabetes mellitus and cerebrovascular disease, and the results were shown in Fig. 4. In addition, the composition of comorbidities of the top 5 diseases were counted. The most common comorbidities of malignant tumor were hypertension (18.52%), diabetes mellitus (10.08%), hepatic disease (9.26%), ischemic heart disease (7.41%) and respiratory disease (6.03%). As for the comorbidities of hypertension, ischemic heart disease (16.79%), malignant tumor (14.58%), other types of heart disease (9.45%), diabetes mellitus (9.07%) and cerebrovascular disease (6.61%) ranked top 5 in the list. The most common comorbidities of ischemic heart disease were hypertension (19.31%), other types of heart disease (13.42%), diabetes mellitus (9.98%), malignant tumor (7.14%) and hyperlipidemia (7.07%). In patients with diabetes mellitus, hypertension (16.62%), ischemic heart disease (15.15%), other types of heart disease (6.39%) and hyperlipidemia (5.03%) were the prevalent comorbidities. When it comes to cerebrovascular disease, hypertension (17.21%), ischemic heart disease (12.87%), other types of heart disease (7.62%), diabetes mellitus (7.13%) and malignant tumor (6.89%) were the top 5 diseases combined.

#### Causes of mortality

**Fig.5:**
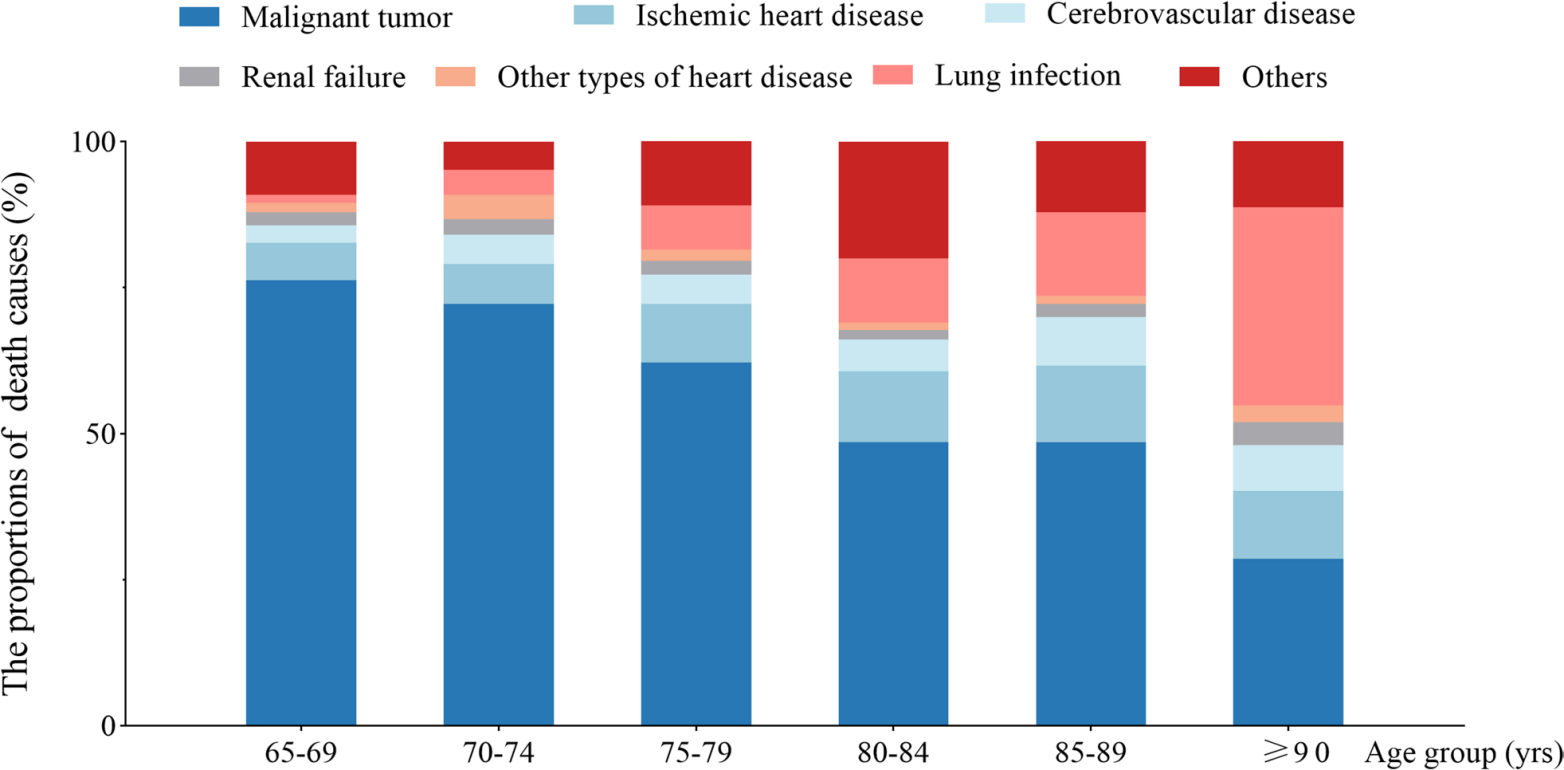
The composition of death causes in various age groups. Lung infection is a common respiratory system disease mainly caused by pathogenic microorganisms, which invade the "lower respiratory tract, alveoli and other lung structures, thereby causing inflammatory reaction [22]

A total of 2521 patients, defined as in-hospital death with complete death records in Chinese PLA General Hospital, were enrolled in the death cause analysis. We counted the proportions of various diseases in the overall sample, among which, malignant tumor accounted for 49.51%, ischemic heart disease 16.41%, cerebrovascular disease 9.34%, lung infection and chronic lower respiratory disease 9.11%, other types of heart disease 3.35%, and other causes accounted for 12.27%. In addition, we further analyzed the compositions of death causes in different age groups and the results were shown in Fig. 5. With the aging of the overall sample in the analysis of death causes, the proportion of patients died of malignant tumor significantly decreased, while that died of lung infection increased. Since malignant tumor accounted for 49.51% of in-hospital death, we analyzed the mortality by cancer site and we found that lung cancer (26.3%), liver cancer (14.5%), stomach cancer (11.9%), colorectal cancer (8.6%), and pancreatic cancer (8.1%), were the most prevalent cancers related to in-hospital death in our study, accounting for 85.7% of the total cancer deaths. The results are shown in Figure S3.

### The status of polypharmacy in the study population

**Fig.6:**
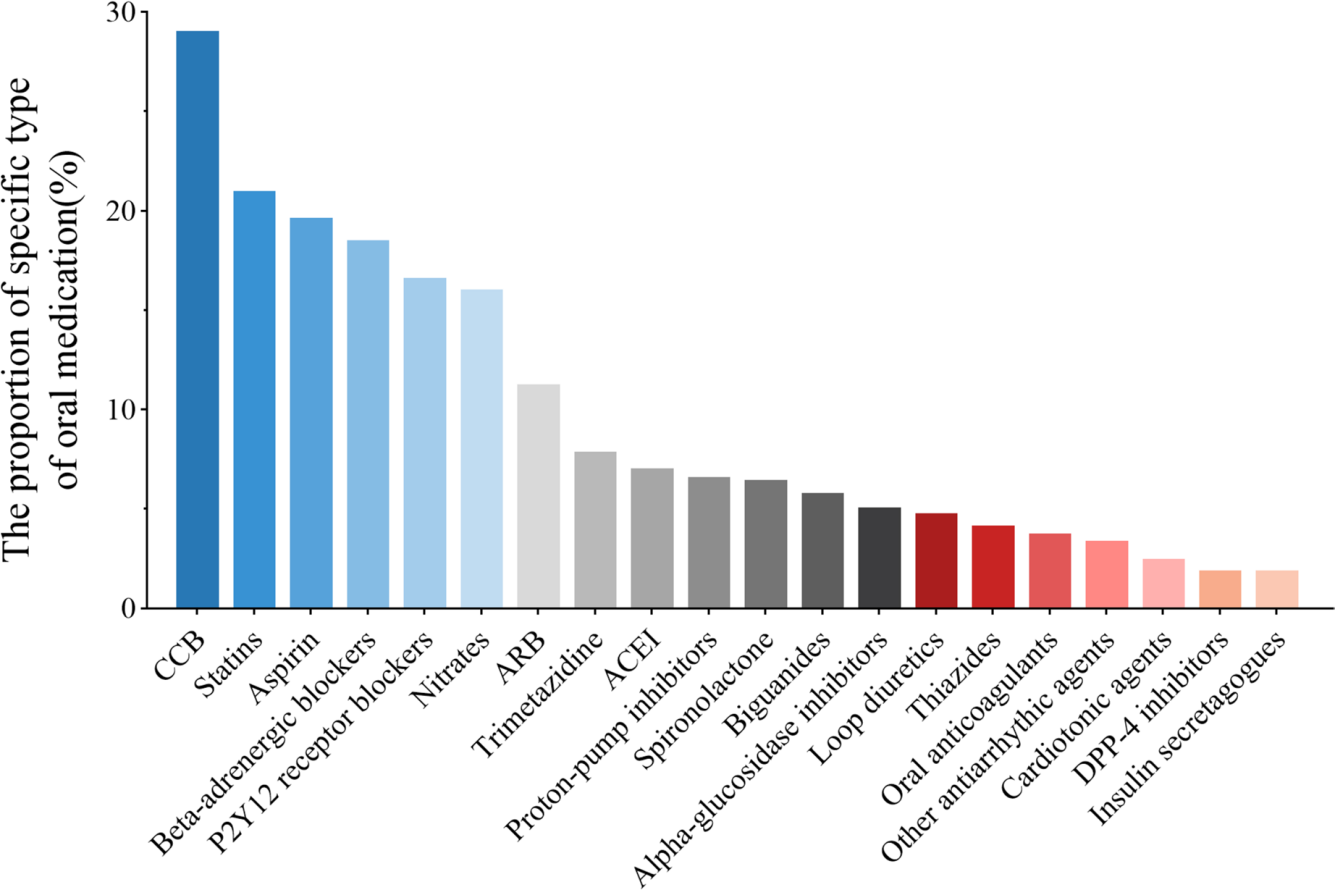
The ranking and proportion of oral medications in the study population. CCB: Calcium channel blocker; ARB: Angiotensin Receptor Blocker; ACEI: Angiotensin Converting Enzyme Inhibitor; DPP-4: Dipeptidyl peptidase-4

The average number of oral medications taken by the study population was 5.4 types, and the proportion of patients taking more than 5 kinds of medications accounted for 55.42%, while those taking one kind accounted for only 12.56%. Later, we counted various kinds of oral medications taken by the study population. The results were shown in Fig. 6, indicating that calcium channel blockers (29.02%), statins (21.00%), aspirin (19.63%), β-blockers (18.52%), P2Y12 receptor antagonists (16.61%), nitrates (16.05%), angiotensin receptor blocker (11.27%), Trimetazidine (7.89%), angiotensin-converting enzyme inhibitor (7.06%) and proton-pump inhibitors (6.62%) were the top 10 medications taken by the study population.

## Discussion

In this study, we retrospectively analyzed the disease composition, the status of multimorbidity and polypharmacy of the elderly inpatients aged ≥ 65 years old in Chinese PLA General Hospital from January 2008 to December 2019. The average age of the study population was 72.67 years old. We counted the constitution of common diseases and multimorbidity based on items of discharge diagnoses. The results showed that malignant tumor ranked high among the study population, followed by hypertension, ischemic heart disease, diabetes mellitus and cerebrovascular disease, which were similar to the results of previous global studies on the burden of chronic diseases. A previous study pooled a total of 90,758 subjects derived from 75 articles and found that hypertension, hyperlipidemia, diabetes mellitus, heart disease, cerebrovascular disease and COPD are the most prevalent diseases in Chinese officers aged 60 years and over [23]. The fine distinction between our study and the cited article is mainly due to the difference in the study population. We focused on elderly inpatients, who are more likely to be hospitalized for malignant tumor, while administration staff are more likely to seek medical services in outpatient clinics instead of undergoing inpatient therapy for the management and follow-up of chronic diseases. It is also worth noting that hypertension, ischemic heart disease, diabetes mellitus and cerebrovascular disease are most prevalent in both studies. Therefore, it is in urgent need to carry out primary or secondary prevention and early screening of cardio-cerebrovascular diseases for the elderly whether in community population or hospitalized inpatients. For elderly patients who have been diagnosed with common chronic diseases, it can be helpful to strengthen the management of these diseases, emphasizing the monitoring and controlling of blood pressure, glucose, lipid and other risk factors related to common chronic diseases. In recent years, a large number of studies have focused on life habits which are considered to be associated with cardiovascular diseases, metabolic diseases and other common chronic diseases in the elderly. Physical activity, sedentary behavior and weight control are the most concerned factors. A recent study revealed that the risk of type 2 diabetes mellitus (T2DM) could be significantly reduced with only six minutes of moderate-to-vigorous physical activity. In addition, the longer the duration of exercise, the lower the risk of T2DM [24]. As for weight control, previous studies suggested an association between the magnitude of weight loss and the incidence of cardiovascular disease in people with type 2 diabetes [25].

The proportion of malignant tumor in aged and super-aged elderly inpatients exhibited a declining trend. This finding could be due to the following reasons: Firstly, the overall survival of malignant tumor is less than other chronic diseases such as hypertension and respiratory diseases, and a large portion of elderly patients diagnosed with cancer can not survive over 80–85 years old. Secondly, aged and super-aged patients with malignant tumor are more likely to rehabilitate at home instead of being hospitalized because of poor physical condition, more and severe complications, as well as reduced willingness [26]. Additionally, we focused on the diseases of which the proportion presenting an overall upward trend in both male and female patients, including other types of heart disease and respiratory disease. In this part, the other types of heart disease referred to various types of arrhythmia, heart failure, cardiomyopathy, cardiac valvular disease, cardiac death and other heart diseases except for ischemic heart disease which has been clarified separately. Since the compensatory and reserve function of organs tends to deteriorate with age, the elderly are much more prone to develop complications due to primary diseases. Therefore, the proportions of various heart diseases, such as secondary arrhythmia, primary and secondary heart failure showed upward trends in general. When it comes to respiratory disease, we analyzed common respiratory diseases in the elderly inpatients, including chronic obstructive pulmonary disease, emphysema, bronchiectasis, asthma, bronchitis, and pneumonia. Based on the analysis of specific categories, we found that the proportion of pneumonia significantly elevated with age. It is widely acknowledged that pneumonia is one of the leading causes of morbidity and mortality in elderly patients. The increased frequency of pneumonia can be explained by the physiological changes linked to the progressive aging of the respiratory tree and the diminished immunological response [27]. Therefore, we should be vigilant about the occurrence of multiple complications, assess the risk in the early stage and take active measures to prevent complications. For example, we should pay attention to body position to avoid aspiration, and strengthen oral care to reduce the risk of pneumonia for elderly inpatients.

When it comes to the causes of hospitalization, we found that malignant tumor and ischemic heart disease still ranked high, while other diseases were not mentioned in the list of top 10 diseases, such as arthropathy, lens disease, and biliary and pancreatic disease in males, as well as arthropathy, lens disease, and injuries to hip and thigh in females. Therefore, we can infer that although the incidence of diabetes mellitus and hypertension is relatively high among the elderly inpatients, a large number of patients were admitted to hospitals with arthropathy and lens disease rather than such stable chronic diseases. The proportion of arthropathy showed a downward trend while injuries to hip and thigh presented an upward trend in female inpatients. According to previous reports, the onset of osteoarthropathy in females was most common at the age of 40 to 65, which may be related to the decline in the proportion of patients hospitalized for arthropathy with age in our study [28]. According to statistics, about 20 million elderly people in China have at least one fall every year, with direct medical costs exceeding 5 billion RMB. Meanwhile, falls have become the leading cause of injury-related death in the elderly [29, 30]. The most common injury associated with falls in the elderly is osteoporotic fracture [31]. Injuries of hip and thigh is mostly found in elderly patients aged over 72 years, with more than 90% attributable to falls, which is regarded as the leading cause of hospitalization after falls. It is worth noting that the prevalence of falls in females is significantly higher than in males. The underlying reason is osteoporosis caused by the decrease of estrogen levels in postmenopausal females, which is strongly related to an elevated incidence of falls, contributing to the increased risk of hip and thigh injuries [32]. A large number of elderly patients hospitalized for hip and thigh injuries are prone to serious complications such as pulmonary infections and deep vein thrombosis because of prolonged recumbency. Injuries of hip and thigh and related complications greatly increase the burden of diseases among elderly female inpatients.

In this study, the number of elderly inpatients with two or more chronic diseases accounted for 91.89%, which was similar to a foreign study, suggesting that the proportion of multimorbidity among the elderly could be as high as 95.1% [33]. We have found that malignant tumors have the highest burden of > 5 comorbidities, which was shown in Fig. 4. In view of the cancer statistics, lung cancer and liver cancer are the most prevalent in China [34]. According to the data of malignant tumor sites in the analysis of death causes, we can roughly guess that lung cancer and liver cancer are the most prevalent sites of malignant tumors in this study, so the proportions of patients combined with liver diseases and respiratory diseases are relatively high, while in patients with no malignant tumor, including patients with hypertension, ischemic heart disease, cerebrovascular disease and diabetes, the proportion combined with liver diseases and respiratory diseases are relatively low. Among these patients, the diseases combined are mostly cardiovascular, cerebrovascular diseases and metabolic diseases which are common in the whole elderly population, which may be one of the reasons for the high number of complications in patients with malignant tumor. Taking lung cancer patients as an example, previous studies have revealed that many patients develop respiratory diseases before the diagnosis of lung cancer, among which, COPD is the most common comorbidities of lung cancer, and it is associated with a significant increase in the incidence of lung cancer. A study indicated that the incidence of combined lung cancer and COPD is as high as 52% [35]. Besides, we believe that it can be attributed to the underlying diseases distribution of the study population, the organ injuries such as malnutrition, infection, diabetes, kidney dysfunction caused by cancer itself, adverse reactions to antitumor therapy, drug cardiotoxicity and so on.

We have analyzed the most prevalent types of multimorbidity and have noticed that the patients with combined malignant tumor and hypertension accounted for a large proportion. A previous study suggested that hypertension is the most common comorbidity in elderly patients with malignant tumor, with an incidence of 38% [36]. Besides, a cohort study in Canada indicated that among 6000 cases of survived cancer patients, 43% developed hypertension. Except for hypertension, heart failure and other cardiac conditions were associated with cancer. Studies have shown that patients with cancer have an 8.2-fold increased risk of cardiac death, and long-term cancer survival patients have a 15-fold increased risk of heart failure [37–39]. The possible reasons were listed as follows: Firstly, cancer and hypertension are both common diseases due to the aging of the population. Secondly, with the advances in medical technology, the survival rate of cancer patients obviously elevated in recent years [40]. Thirdly, several risk factors of hypertension, such as obesity, smoking and a sedentary lifestyle, are also related to the development of cancer [41]. Additionally, previous studies have shown that cancer survivors had a substantially higher risk of hypertension and heart failure, which might be attributable to the exposure to cardiotoxic chemotherapy agents in cancer patients, such as anthracyclines,5-fluorouracil, paclitaxel, erythritol-elastic oncogene B-2 (ErbB2) and other types of chemotherapy agents, which cause injury to endothelial cells and cardiomyocytes, eventually leading to the development of hypertension, heart failure and even cardiac death [42, 43]. This might be one of the crucial reasons explaining the mechanism of cancer-related hypertension and other cardiovascular diseases. When it comes to the interactions between cancer and hypertension, both malignant tumor and hypertension are proliferative and inflammatory diseases, thus promoting each other and progressing rapidly [44]. Secondly, hemodynamic changes and the features of being prone to form thrombosis caused by hypertension accelerate tumor progression and metastasis [45]. To sum up, there is plenty of evidence supporting the high prevalence of combined malignant tumor and hypertension. In addition, it is in urgent need for oncologists and cardiologists to raise awareness of cardiovascular complications in cancer patients. A comprehensive and patient-targeted strategy might be helpful with cardiovascular health maintaining, as well as a better prognosis in cancer patients.

For elderly patients with multimorbidity, we need to pay attention to the general condition and the status of multi-system diseases. Moreover, the elderly are susceptible to various diseases because of the reduced reserve capability and compensatory function of the organism, eventually leading to multi-organ dysfunction [46]. The number of patients with heart failure complicated with renal failure ranked high, accounting for more than 50%. Firstly, a previous study showed that the number of patients with chronic kidney disease (CKD) is increasing worldwide, and CKD has been regarded as a global epidemic enhanced by increasing rates of diabetes and hypertension. In turn, hypertension and diabetes were associated with renal failure and heart failure, which has been found as a complication of advanced renal disease [47]. Data obtained from the death registration system of the National Health Commission of China revealed that circulatory, cancer, respiratory disease and diabetes caused most of the chronic disease-associated deaths. Which was partially consistent with the findings in our study. Therefore, we believe that it is crucial to pay more attention to the elderly, closely monitor the indicators of organ function, and identify high-risk patients, so as to realize timely attention, evaluation and intervention, with the ultimate goal of improving the outcome.

In terms of medication, the top 10 drugs used in the study population were antihypertensive, cholesterol-lowering drugs and antiplatelets. Calcium channel blockers are the most commonly used drugs in elderly patients with hypertension, followed by β-blockers, angiotensin receptor inhibitors, and angiotensin-converting enzyme inhibitors. Antiplatelets including aspirin and P2Y12 receptor antagonists (clopidogrel bisulfate + ticagrelor) are also commonly used in elderly inpatients. It is worth noting that the study population took an average of 5.4 kinds of drugs, and over 50 percent of patients took more than 5 kinds of medications. Polypharmacy is often defined as the long-term use of more than five kinds of prescribed drugs daily [48]. In clinical practice, polypharmacy might be reasonable and beneficial for patients with multimorbidity. However, the risk of inappropriate prescribing related to polypharmacy significantly increased, leading to adverse outcomes [18]. Therefore, it is necessary to pay attention to the adverse effects of multiple drugs, especially in patients with multimorbidity, such as the cardiotoxicity of antitumor agents, and bleeding disorder associated with antiplatelet drugs during the perioperative period, etc. Consequently, multi-disciplinary treatment (MDT) is essential for elderly patients with multimorbidity, and an individualized comprehensive therapeutic strategy should be developed, so as to avoid the risk of adverse effects caused by inappropriate prescription and eventually improve outcomes.

Our investigation should be interpreted in the context of several limitations. Firstly, our study was designed as a baseline full-sampled survey without standardized follow-up and could not provide supporting evidence on the risk of diseases, nor on causal relationships. Secondly, the data were obtained from a single center and only inpatients were enrolled in our study. Besides, we have not included data since the outbreak of COVID-19. Therefore, it would be beneficial to collect follow-up data in future study. If permitting, we are planning to get clinical data of inpatients and outpatients from multiple clinical centers, so as to make the reported results more representative and universal. Meanwhile, it would be meaningful to analyze the variations of common diseases and multimorbidity before and after COVID-19 to figure out the impact of COVID-19 on diseases spectrum. Despite the limitations listed above, it is a large-scale full-sampled survey over 12 years, including diseases spectrum, multimorbidity, polypharmacy and cause of death in the past decade, providing supporting evidence for reasonable treatment strategy in the elderly inpatients. Meanwhile, we focused on elderly inpatients, who are likely to develop more comorbidities and have a worse prognosis compared to the elderly in community or outpatient clinics, distinguished from most of the previous studies.

In this study, we analyzed the disease spectrum including common diseases and multiple organ failures, the profile of multimorbidity, death causes and oral medications among the elderly inpatients in the past decade. It is suggested that we should adopt active measures to prevent the development and progression of multiple chronic diseases in the elderly, pay attention to the comprehensive management of common chronic diseases such as malignant tumor, hypertension, ischemic heart disease and diabetes mellitus, strengthen MDT, and formulate rational drug use plan and treatment strategy for elderly patients with multimorbidity.

### Supplementary Information

**Additional file 1: Fig. S1.** The age composition of the study population.

**Additional file 2: Fig. S2.** The composition of multimorbidity in the study population. The proportions of patients with various numbers of diseases were counted, among which, patients with two or more chronic conditions were recognized as patients with multimorbidity. The proportion of multimorbidity in the study population was as high as 91.89%.

**Additional file 3: Fig. S3.** The proportion of elderly inpatients died of malignant tumors at different sites.

**Additional file 4: Table S1.** The composition of single and two types of organ failure in the study population.

## Data Availability

Data are available on request from the corresponding authors, Yabin Wang and Feng Cao, upon reasonable request.
