# Supplementary figures and images for: Chronic diseases spectrum and multimorbidity in elderly inpatients based on a 12-year epidemiological survey in China
Source: BMC Public Health. 2024 Feb 17;24:509. doi: 10.1186/s12889-024-18006-x (PMC10874035; doi:10.1186/s12889-024-18006-x)

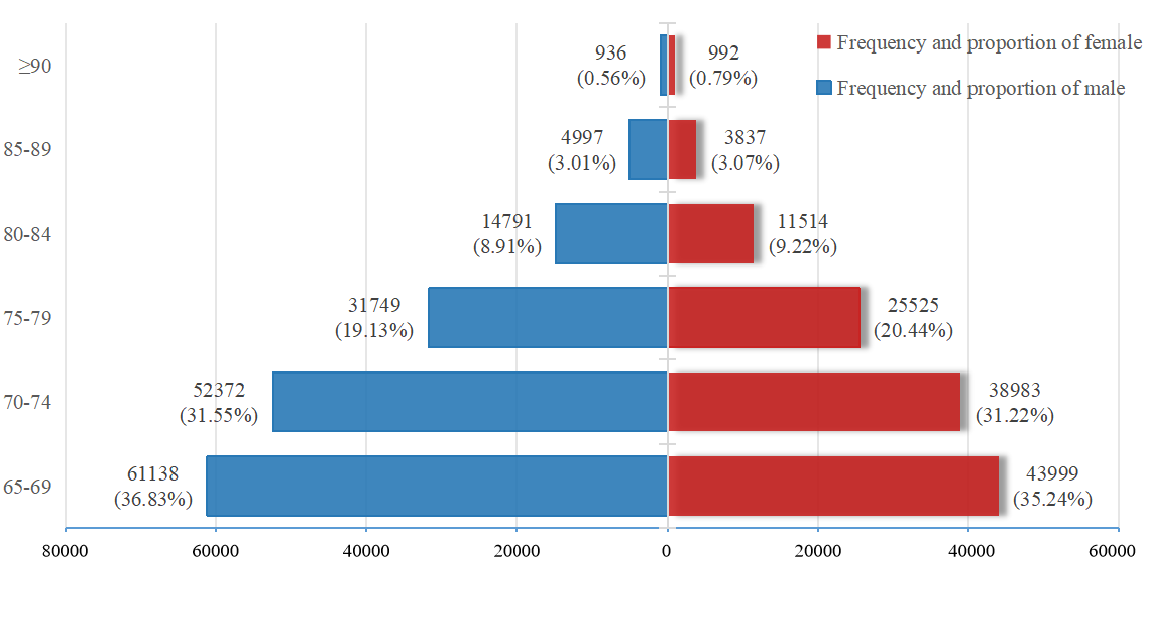

Supplement: Supplementary file 1 — Additional file 1: Fig. S1. The age composition of the study population. [file 12889_2024_18006_MOESM1_ESM.tif]

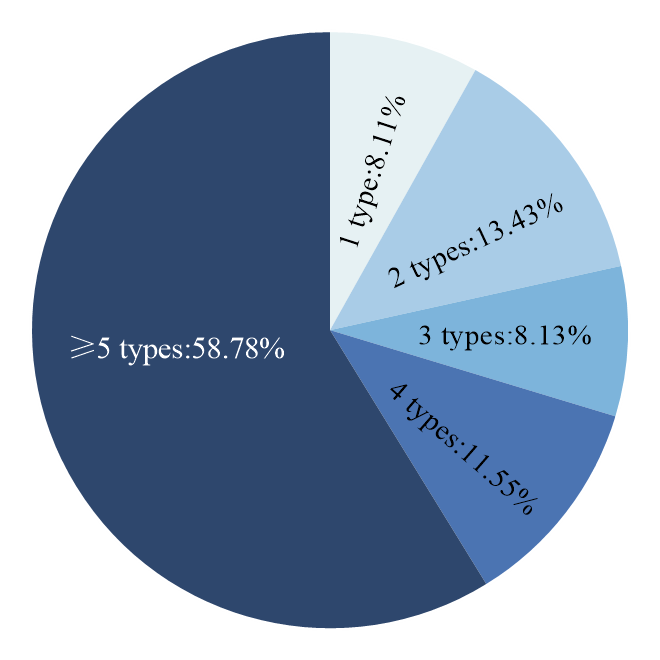

Supplement: Supplementary file 2 — Additional file 2: Fig. S2. The composition of multimorbidity in the study population. The proportions of patients with various numbers of diseases were counted, among which, patients with two or more chronic conditions were recognized as patients with multimorbidity. The proportion of multimorbidity in the study population was as high as 91.89%. [file 12889_2024_18006_MOESM2_ESM.tif]

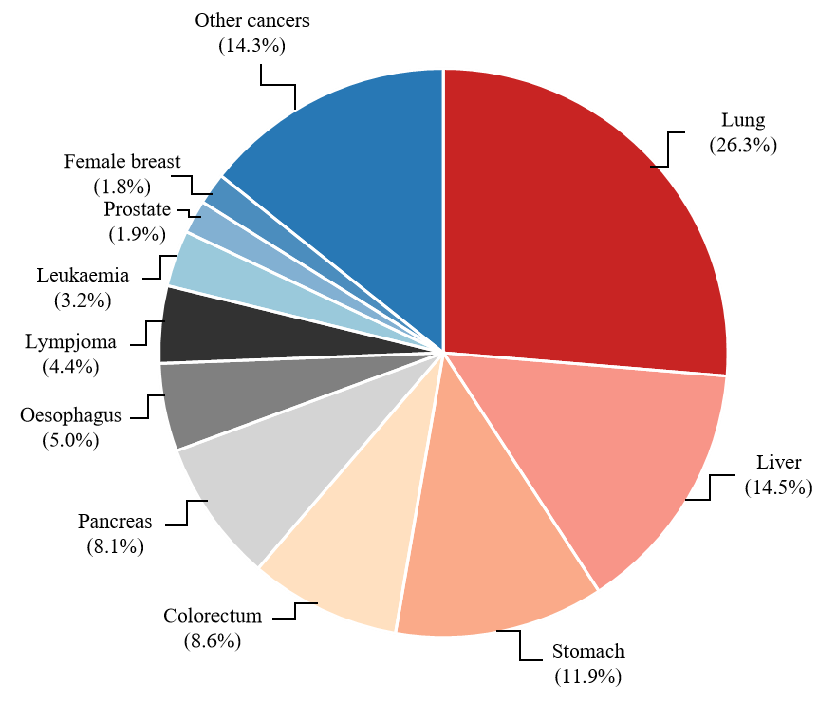

Supplement: Supplementary file 3 — Additional file 3: Fig. S3. The proportion of elderly inpatients died of malignant tumors at different sites. [file 12889_2024_18006_MOESM3_ESM.tif]
