# Supplementary material for: Chronic diseases spectrum and multimorbidity in elderly inpatients based on a 12-year epidemiological survey in China
Source: BMC Public Health. 2024 Feb 17;24:509. doi: 10.1186/s12889-024-18006-x (PMC10874035; doi:10.1186/s12889-024-18006-x)
Supplement: Supplementary file 4 — Additional file 4: Table S1. The composition of single and two types of organ failure in the study population. [file 12889_2024_18006_MOESM4_ESM.docx]

TableS1. The composition of single and two types of organ failure in the study population

| Items | Frequency (Proportion) |
| --- | --- |
| Single organ failure | |
| Heart failure | 3293 (41.36%) |
| Renal failure | 3406 (42.77%) |
| Respiratory failure | 1030 (12.93%) |
| Hepatic failure | 234 (2.94%) |
| Two types of organ failure | |
| Heart failure + Renal failure | 2857 (53.76%) |
| Heart failure + Respiratory failure | 946 (17.81%) |
| Respiratory failure + Renal failure | 631 (11.87%) |
| Hepatic failure + Renal failure | 352 (6.63%) |
| Heart failure + Hepatic failure | 248 (4.67%) |
| Respiratory failure + Hepatic failure | 281 (5.26%) |

All data were counted according to the number of hospitalizations. Data was given as proportions unless otherwise indicated. Proportions were calculated using the total number of each item as the denominator.
